# Causal associations between blood pressure and the risk of myocardial infarction: A bidirectional Mendelian randomization study

**DOI:** 10.3389/fcvm.2022.924525

**Published:** 2022-11-10

**Authors:** Zhi-Qiang Yang, Ting-Ting Fan, Zheng Wang, Wan-Ting Zhou, Zhen-Xian Wang, Yan Tan, Qi Wu, Bang-Long Xu

**Affiliations:** Department of Cardiovascular Medicine, The Second Affiliated Hospital of Anhui Medical University, Hefei, China

**Keywords:** blood pressure, myocardial infarction, bidirectional Mendelian randomization, genome-wide association study, causation

## Abstract

**Introduction:**

Many observational studies imply elevated blood pressure (BP) as a leading risk factor for incident myocardial infarction (MI), but whether this relationship is causal remains unknown. In this study, we used bidirectional Mendelian randomization (MR) to investigate the potential causal association of BP levels with the risk of MI.

**Methods:**

Genetic variants associated with BP and MI traits were retrieved from the International Consortium of Blood Pressure (*N* = 7,57,601) and UKB (*N* = 3,61,194), obtaining 1,26,40,541 variants. We used two-sample MR (TSMR) analyses to examine the potential bidirectional causal association of systolic BP (SBP), diastolic BP (DBP) and pulse pressure (PP) with MI.

**Results:**

The forward MR analysis identified a potentially causal association between MI and BP except PP[odds ratio (OR) _SBP_: 1.0008, *P* = 1.911 × 10^−22^; OR_DBP_: 1.0014, *P* = 1.788 × 10^−28^;odds ratio (OR)_pp_: 1.0092, *P* = 0.179]. However, the reverse analysis suggested no causal relation (beta_SBP_: 5.469, *P* = 0.763; beta_DBP:_ 3.624, *P* = 0.588; beta_PP:_ −0.074, *P* = 0.912). These findings were robust in sensitivity analyses such as the MR–Egger method, the maximum likelihood method and the MR pleiotropy residual sum and outlier test (MR-PRESSO). No horizontal pleiotropy (*p* = 0.869 for SBP, *p* = 0.109 for DBP and *p* = 0.978 for PP in the forward results and *p* = 0.168 for SBP, *P* = 0.892 for DBP and *p* = 0.989 for PP in the reverse results) was observed.

**Conclusions:**

Elevated SBP or DBP levels increase the risk of MI, but there is no causal relationship between MI and changes in BP including PP. Independent of other risk factors, optimal BP control might represent an important therapeutic target for MI prevention in the general population.

## Introduction

Hypertension (HT) and myocardial infarction (MI) are two interconnected global public health burdens. The estimated prevalence of hypertension is 31%, and coronary artery disease (CAD) affects 10% of adults (1). Hypertension, which is a modifiable, independent cardiovascular risk factor and a major global public health burden, accelerates the atherosclerotic process. Sustained high blood pressure (BP) also alters the myocardial structure due to fibrosis and myocyte hypertrophy (2–4).

Myocardial infarction, a major reason of mortality and morbidity in older adults, is a term for a heart attack event that originates from the Latin: infarctus myocardii, or MI (5). When one of the coronary arteries that supplies blood to the cardiac muscle is blocked by an embolus, such as plaques, white blood cells and fat, the epicardium is the first tissue to undergo ischaemia because of a lack of oxygen supply, and necrosis of the heart muscle occurs. Hence, effective prevention of MI is critical, as it might obviously improve quality of life and lower the mortality rate.

Among the risk factors, age has the strongest relationship with the development of CAD and myocardial infarction (6, 7). Other risk factors for MI have been attested from large studies about longitudinal cohort and include hypertension, alcohol, cigarette smoking, obesity, sex, and diabetes (3, 7, 8). A history of HT is a common risk factor among patients with MI. Sarah et al. (9) reported that mean BP and variability in BP are associated with cardiovascular outcomes. Previous studies have shown that systolic and diastolic blood pressure are closely and directly associated with CAD mortality at all ages and that lowering BP can rapidly reduce heart disease risk (6). These studies, however, are inclined to systematic biases such as statistical and clinical methodological problems and cannot support a causality between high BP and the risk of MI.

It is difficult to confirming a causality, as the effect between BP and MI might be confounded with several disparate factors. For instance, elevated BP is usually associated with advanced age, which is also an vital risk factor. This makes it challenging to clarify whether or not BP and MI are related to each other or simply mean comorbidities clustered in older subjects. Mendelian randomization (MR) (10) is an instrument that uses genetic variants as instrumental variables (IVs) of the exposure to estimate the causal effects of the exposure on outcome and can overcome the confounding existing in the observational studies (11). Due to the allocation of genes randomly from parents to their offspring at conception, IVs are less susceptible to confounding or reverse the causality (12, 13). Two-sample MR (TSMR) analyze is an extension of the MR approach that allows the application of summary statistics of genome-wide association studies (GWASs) for MR studies without direct analysis of individual data. The causation risk reversely is minimized too, on the grounds that the history of a disease could not have an effect on an individual's genotype (10). In our study, we carried out bidirectional MR analyses by performing summary-level data from the available GWASs on BP and MI to investigate the role of BP levels in MI causally.

## Method

### Data sources

**Figure 1:**
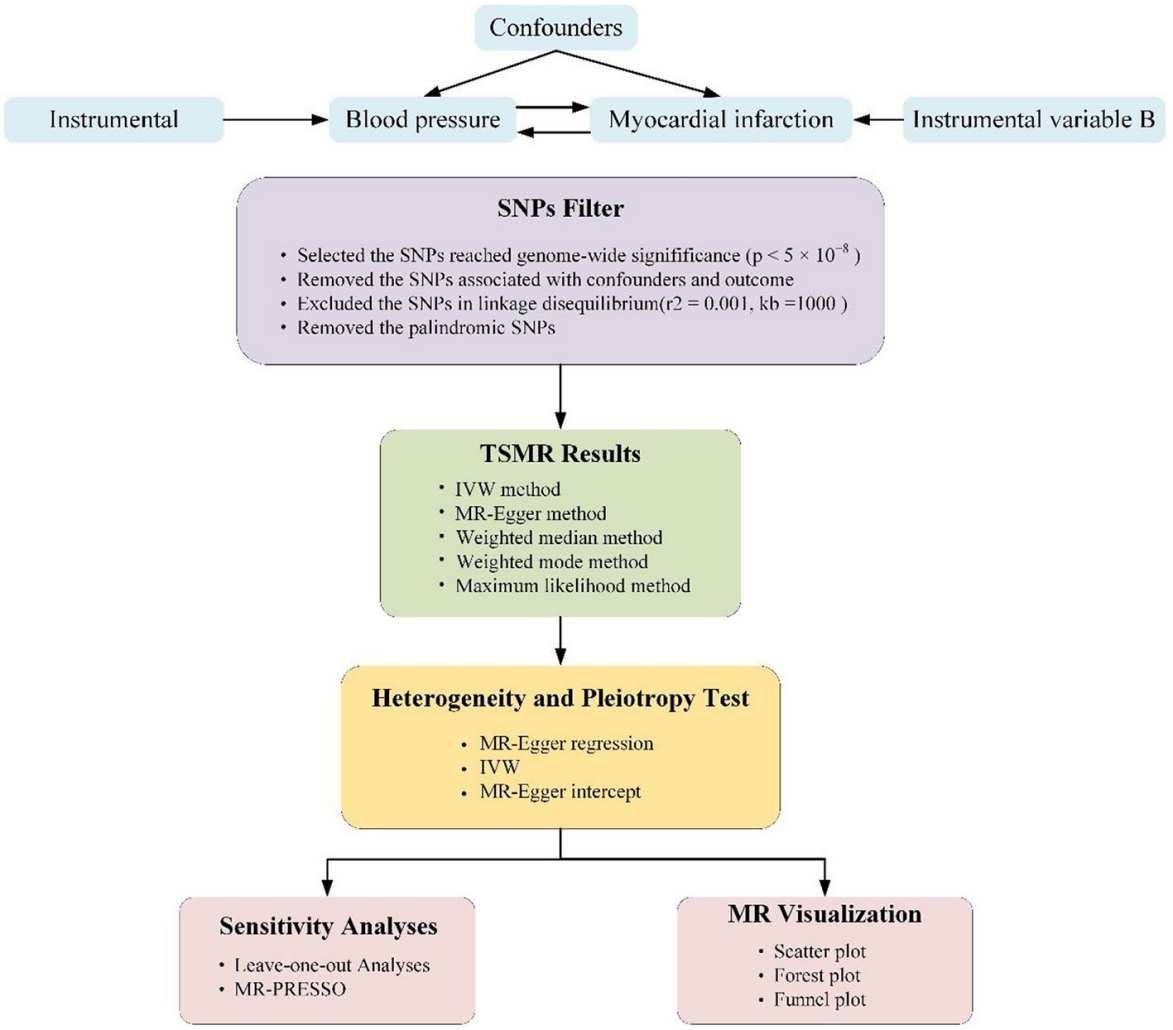
Schematic representation of two-sample Mendelian randomization (TSMR) analysis of our study. The three core assumptions of Mendelian randomization (MR) analysis are as follows: (1) instrumental variables (IVs) must be associated with blood pressure (BP) or myocardial infarction, (2) IVs must not be associated with confounders, and (3) IVs must influence outcome only through exposure.

The analyses conducted in our study were based on public available summary data originated from GWAS group. And, genetic variants related with BP values were performed as IVs for the MR analyses. The summarized GWAS data were extracted from the IEU OpenGWAS project. Blood pressure data were retrieved from the International Consortium of Blood Pressure Genome-Wide Association Studies (ICBP) (14) and Ishigaki etc., (15). The ICBP established which was aimed at exploring BP genetics and remains one of the largest available resources to date. The MI data were acquired from the UKB (*N* = 3,61,194). The detail of the data were listed in Supplementary Table 1. The step-by-step workflow of this study is showed in Figure 1.

### Selection and validation of IVs

We conducted separate TSMR approaches to probe the causal associations potentially between systolic BP (SBP) and diastolic BP (DBP) as well as pulse pressure (PP) and the risk of MI, probing the association in two non-overlapping populations. The three vital assumptions underlying the TSMR method are shown in Figure 1 (implementation of the latter two assumptions serves as the definition of independently pleiotropy):

a. The genetic variants must be closely related to the exposure;
b. The variants must have an effect on the outcome uniquely via their influence on the exposure;
c. The variants ought to be unique of any bias of the affect between the outcome and the exposure (11).

IVs must to be related to the exposure. In our forward MR analysis, the *p*-value of IVs had to be < 5 × 10^−8^ in the GWAS data to guarantee a strong relationship between IVs and BP levels. These SNPs were further pruned for linkage disequilibrium (LD; distance threshold = 10,000 kb, *r*2 < 0.001) to guarantee independence among the genetic variants. The single-nucleotide polymorphisms (SNPs) must be removed from our analyses when *r*2 was >0.001. We excluded SNPs associated with acknowledged confounders (such as smoking, high cholesterol levels and obesity). Afterwards, palindromic SNPs were eliminated to guarantee that the effects of the SNPs related to the exposure corresponded to the same allele as their effects on MI.

### Statistical analysis

In our forward MR analysis, we performed the inverse variance-weighted (IVW) approach to calculate the causal effect (16). The fixed-effects IVW method imagines that all SNPs appears no horizontal pleiotropy or other violations of the assumptions (17). The random-effects IVW method loosens the assumption when horizontal pleiotropy is absent, and the variance in this method is exaggerated to in view of the between-SNPs heterogeneity (18). In consideration of the substantial heterogeneity of the effect of the IVs for BP on MI, the horizontal pleiotropy assumption of the fixed-effects IVW method may be invalid. To reduce the impact of potential pleiotropy on the results, we performed weighted median, MR–Egger and weighted mode methods (17, 19, 20). Conducting the MR–Egger method, the effect of each SNP corresponds the exposure was plotted against its role on the outcome, and once pleiotropy did not exist, the plotted points drawn fall along a straight line through the origin. This method requires that no horizontal pleiotropic is related to the SNP-exposure effects (InSIDE assumption) (19).

The weighted median requires that 50% of the weight valued to variables be from valid instruments (19). In contrast, the weighted mode demands the largest subset of instrumental variables that notarize the same one to be effective. We also conducted the maximum likelihood approach, with which the effect was calculated by the likelihood maximization directly in terms of the SNP related to exposure and outcome effects and the assumption of a linearity relationship between the outcome and exposure (21). Be akin to the fixed-effects IVW model, the maximum likelihood approach requires absent heterogeneity or pleiotropy. Compared with the IVW method, other approaches are more stable for individual genes with forceful causal results and produce a consistent result of the causative effect when valid IVs surpass 50% (22). The MR pleiotropy residual sum and outlier (MR-PRESSO) test was performed to clear horizontal pleiotropic outlier variants and provide an outlier-corrected estimate (23). We use the F statistic to assess the effectiveness of the instrumental variables between IVs and exposures. An F statistic >10 was identified as sufficient enough to avert deviation from the causal IV strength evaluate (24).

We performed leave-one-out sensitivity approach to identify whether a single SNP disproportionately controlled the effect. All of the our work were two-sided. All analyses were calculated in R version 4.0.5.All methods were performed using the “TwoSampleMR” and “MRPRESSO” package.

## Results

### Causal effect of blood pressure on myocardial infarction

**Figure 2:**
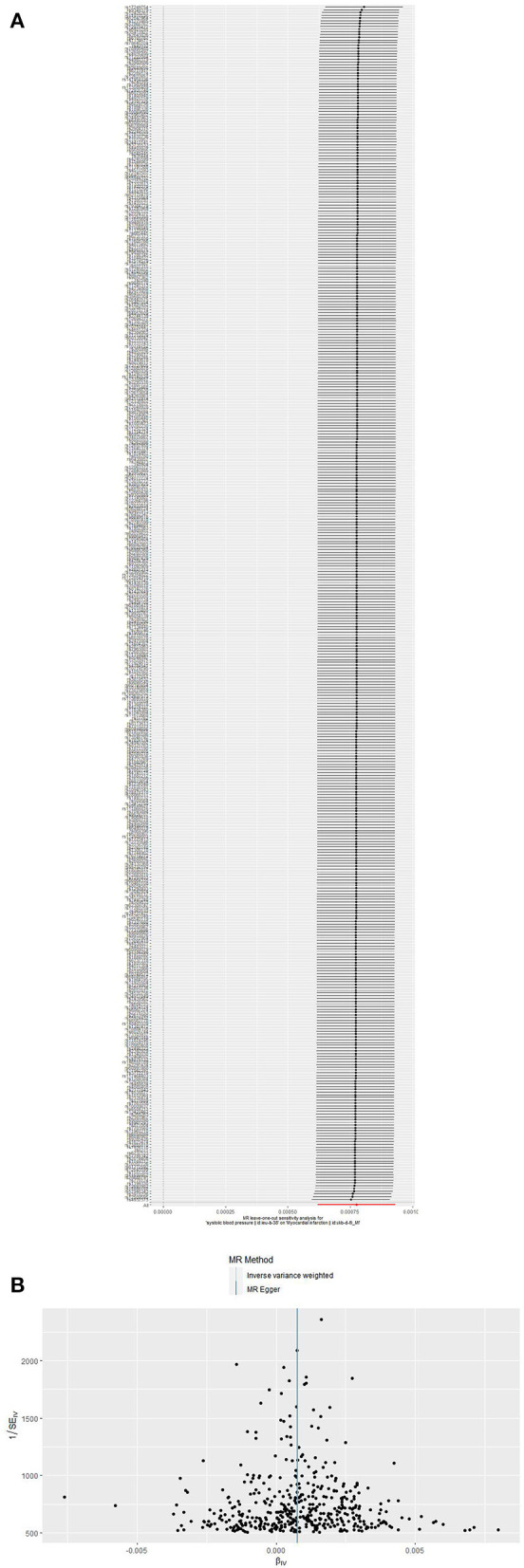
**(A,B)** Leave-one-out sensitivity method and funnel plots in the SBP → MI MR analysis. **(A)** Leave-one-out sensitivity method. Every black point corresponds the IVW method applied to calculate the causal effect of SBP on MI, excluding that particular variant from the analysis. The red point corresponds the IVW estimate using all of the SNPs. There are no instances where the exclusion of one particular SNP leads to dramatic changes in the overall result. **(B)** Funnel plot of the causal association of SBP with MI. Funnel plot showing the causal association of SBP with MI estimated using each SNP as a separate instrument against the inverse of the standard error of the causal estimate. Vertical lines show the causal estimates using all of the SNPs combined into a single instrument for the two different methods. Symmetry in the funnel plot confirms this assumption.

**Figure 3:**
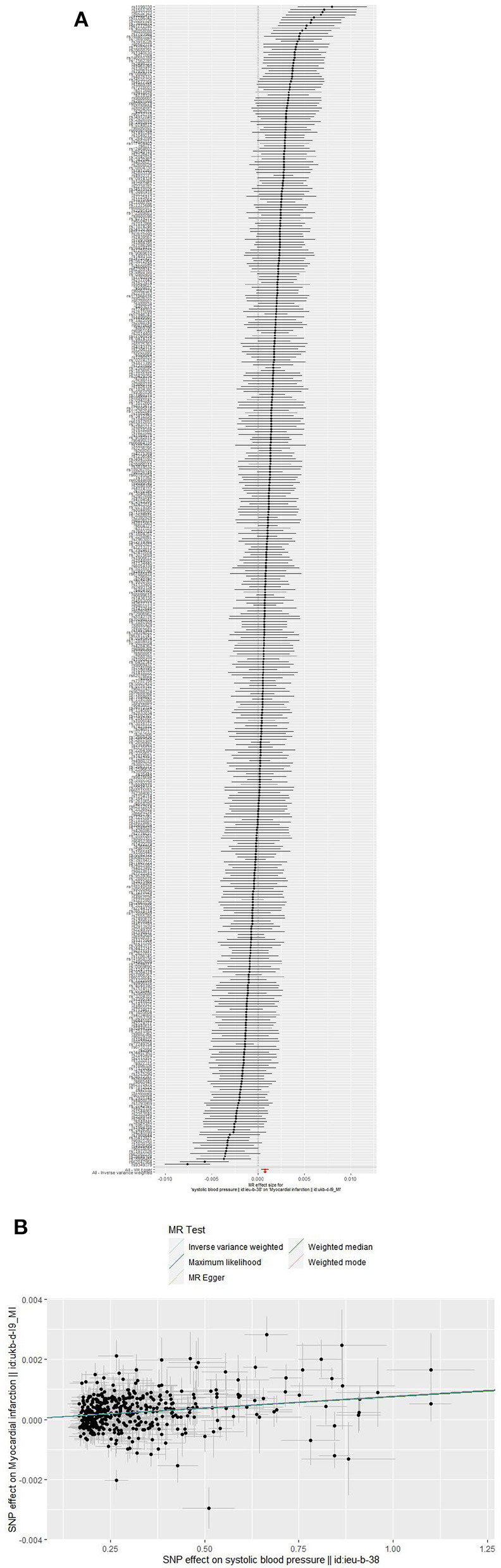
**(A,B)** Forest plots **(A)** and scatter plots **(B)** of causal relationships between SBP-associated SNPs and risk of MI. The slopes of each line in the scatter plots represent the causal association for each method.

After the exclusion of SNPs in LD (*r*2 = 0.001, 10,000 kb), 461 SNPs for SBP,460 SNPs for DBP and 16 SNPs for PP were identified as instruments in the GWAS. After assessment for the three key assumptions of TSMR was conducted and the palindromic SNPs were removed, 434 SNPs for SBP,442 SNPs for DBP and 15 SNPs for PP were included in the analysis. Alcohol consumption, diabetes, smoking, and cholesterol and triglyceride levels were used as confounders. The details of our forward MR analysis are shown in Table 1. A dramatical positive causal result was displayed by IVW and other methods (OR: 1.0008, 95% CI: 1.0006–1.0009, *P* = 1.911e−22 for SBP, OR: 1.0014, 95% CI: 1.0011–1.0016, *P* = 1.788e-28 for DBP and OR: 1.0092, 95% CI: 0.9958–1.0227, *P* = 0.179 for PP). Random effect methods were performed to account for the material heterogeneity exposed in IVW (P_SBP_ = 7.102e-23, P_DBP_ = 9.912e-15,P_pp_ = 8.795e-7) and MR–Egger (P_SBP_ = 5.304e-23, P_DBP_ = 1.716e-14,P_pp_ = 1.850e-6). Notably, the intercept obtained with the MR–Egger approach was un-noteworthy, hinting that the SNPs correspond to BP did not perform any pleiotropic effects (P_SBP_ = 0.869, P_DBP_ = 0.109, P_pp_ = 0.978). The overall estimates revealed causal associations between BP and MI (**Figure 3**, Supplementary Figures 1B, 2, 8). Sensitivity analyses conducting the leave-one-out association approach also verified the results (Figure 2A, Supplementary Figures 1A, 7A). Moreover, funnel plot were asymmetry (Figure 2B, Supplementary Figure 7B).

**Table 1:** Summary of forward MR results of pressure and myocardial infarction.

**Exposure**	**Outcome**	**MR results**					**Heterogeneity**	**Pleiotropy**	
		**Methods**	**n SNPs**	**OR**	**95% CI**	***P*-value**	***P*-value**	***P*-value**	***F* statistic**
SBP	MI	IVW	434	1.0008	1.0006–1.0009	1.911e^−22^	7.102e^−23^		28
		MR–Egger	434	1.0007	1.0004–1.0011	1.976e^−04^	5.304e^−23^	0.869	
		Weighted median	434	1.0008	1.0006–1.0009	6.032e^−14^			
		Weighted mode	434	1.0008	1.0003–1.0013	1.821e^−03^			
		Maximum likelihood	434	1.0008	1.0007–1.0009	1.927e^−38^			
DBP		IVW	442	1.0014	1.0011–1.0016	1.788e^−28^	9.912e^−15^		30
		MR–Egger	442	1.0018	1.0012–1.0024	3.452e^−09^	1.716e^−14^	0.109	
		Weighted median	442	1.0013	1.0010–1.0016	9.956e^−15^			
		Weighted mode	442	1.0012	1.0003–1.0021	7.943e^−03^			
		Maximum likelihood	442	1.0014	1.0012–1.0016	1.122e^−43^			
PP		IVW	15	1.0092	0.9958–1.0227	0.179	8.795e^−7^		14
		MR–Egger	15	1.0079	0.9205–1.1036	0.868	1.850e^−6^	0.978	
		Weighted median	15	1.0103	0.9984–1.0224	0.090			
		Weighted mode	15	1.0112	0.9910–1.0319	0.297			
		Maximum likelihood	15	1.0096	1.0024–1.0168	0.009			

MR, Mendelian randomization; SNP, single-nucleotide polymorphism; OR, odds ratio; CI, confidence interval; IVW, inverse variance weighted; SBP, systolic blood pressure; DBP, diastolic blood pressure; PP, Pulse pressure.

Both the raw and outlier-corrected estimates (excluding 10, 4,3SNPs for SBP, DBP and PP) from the PRESSO approach are consistent to the other results, setting up the association of BP traits with MI on the causal (**Table 3**). We analyzed the *F* values to assess the effectiveness of the relationships among IVs and homologous exposures. The *F* values matching the selected IVs were 28 for SBP, 30 for DBP and 14 for PP, which were efficacious enough to reduce any bias from the results on the causal.

### Causal effect of myocardial infarction on blood pressure

After the exclusion of palindromic SNPs in LD, 7 SNPs for SBP and DBP,5 SNPs for PP were included in the analysis and had an F statistic of 21 and 24, respectively. Three approaches all implied a non-significant causal effect of MI on SBP, every methods revealed a un-significant causal effect of MI on DBP and PP, and the SNPs related to MI did not show any pleiotropy (Table 2, Figure 3B, Supplementary Figures 4, 5B, 6, 9B, 10). The estimation calculated by IVW and MR-PRESSO did not disclose associations of MI with BP (Table 3). Sensitivity analyses performing the leave-one-out association approach also emerged the absence of correlations (Supplementary Figures 3A, 5A, 9A).

**Table 2:** Summary of reverse MR results of pressure and MI.

**Exposure**	**Outcome**	**MR Results**					**Heterogeneity**	**Pleiotropy**	
		**Methods**	***n* SNPs**	**beta**	**SE**	***P*-value**	***P*-value**	***P*-value**	***F* statistic**
MI	SBP	IVW	7	5.469	18.158	0.763	1.592e^−19^		21
		MR–Egger	7	57.516	36.144	0.172	5.479e^−13^	0.168	
		Weighted median	7	20.961	6.145	0.001			
		Weighted mode	7	21.017	6.090	0.014			
		Maximum likelihood	7	6.815	5.032	0.176			
	DBP	IVW	7	3.624	6.684	0.588	2.269e^−07^		21
		MR–Egger	7	1.551	16.230	0.928	7.898e^−08^	0.892	
		Weighted median	7	−1.068	4.113	0.795			
		Weighted mode	7	−3.957	4.069	0.368			
		Maximum likelihood	7	3.981	2.671	0.136			
	PP	IVW	5	−0.074	0.630	0.906	0.195		24
		MR–Egger	5	−0.090	1.287	0.949	0.109	0.989	
		Weighted median	5	−0.148	0.578	0.798			
		Weighted mode	5	−0.161	0.612	0.805			
		Maximum likelihood	5	−0.075	0.515	0.884			

MR, Mendelian randomization; SNP, single-nucleotide polymorphism; IVW, inverse variance weighted; SBP, systolic blood pressure; DBP, diastolic blood pressure; PP, pulse pressure; SE, standard error.

**Table 3:** MR-PRESSO estimates between pressure and myocardial infarction.

		**Raw estimates**				**Outlier-corrected estimates**			
		***N***	**OR**	**95% CI**	***P*-value**	***N***	**OR**	**95% CI**	***P*-value**
Forward results	SBP	434	1.0008	1.0007–1.0010	3.101e^−22^	424	1.0008	1.0007–1.0010	1.212e^−27^
	DBP	442	1.0014	1.0011–1.0016	2.909e^−25^	438	1.0014	1.0011–1.0016	2.703e^−26^
	PP	15	1.0092	0.9958–1.0227	0.201	12	1.0072	0.9961–1.0184	0.233
		***N***	**Beta**	**SD**	***P*** **-value**	***N***	**Beta**	**SD**	***P*** **-value**
Reverse results	SBP	7	5.469	18.158	0.773	4	5.286	10.383	0.646
	DBP	7	3.624	6.684	0.607	4	2.953	4.415	0.551
	PP	5	−0.074	0.630	0.912	5	NA	NA	NA

CI, confidence interval; OR, odds ratio; MR-PRESSO, Mendelian randomization pleiotropy residual sum and outlier; SD, standard deviation.

## Discussion

In our TSMR analysis, we demonstrated that BP had a strong causal effect on the high risk of MI through this bidirectional MR methods. It is important that the association between BP and the risk of MI also involved DBP except PP. On the contrary, a causal effect of MI on BP was not confirmed across MR methods. Because there is no fixed regression between systolic and diastolic blood pressure, the results for pulse pressure may not be ideal. These statistical data stresses the necessity of optimal control range in the HT population for preventing MI. Therapeutics for HT population are likely to effectively prevent MI.

Epidemiological findings have stressed the associations strongly between some risk factors for cardiovascular disease and the risk of MI. HT is one of the most common and strong risk factors related to the occurrence of MI (5). Interestingly, a meta-analysis in 2014 by Thomopoulos et al. (25) showed that more intense BP lowering failed to effectively lower the risk of cardiovascular death.

However, their meta-analysis in 2016 about randomized trials indicated that intensive BP control significantly reduces coronary events and cardiovascular mortality (26). Randomized controlled trials showed that lowering SBP to a few mmHg below 130 lowers the risk of acute events, suggesting that patients with a lower initial SBP had a lower MI risk. HT treatment guidelines propose that BP be reduced to 140/90 mmHg and < 130/80 mmHg in high risk patients. Lee et al. found a slight reduction in the relative risk of common heart events among patients who achieved strict SBP treatment levels. However, an intensive SBP level achieved by excessively lowering SBP can significantly increase the risk of low BP and acute adverse vascular events (27). Strict BP control targets may further impair organ perfusion in patients with cardiovascular disease. Nevertheless, another study found that an excessive reduction in BP did not provide additional benefits but did not elevate the risk of MI; this study failed to reveal a strong association about the decrease values of BP and the prevention of MI with a J-shaped model (28).

Similarly, studies found that more frequent achievement of BP targets did not increase cardiac protection (29). On the contrary, Bangalore revealed that BP levels in population with CAD and cardiovascular events followed a J-shaped curve and that a low BP below the threshold (< 110–120/60–70 mmHg) indicated increased mortality rates, which implied that we should adopt appropriate BP targets (30). Moreover, the results revealed that a higher SBP was associated with lower mortality and that a lower DBP was associated with increased mortality (31). By performing MR, we now master powerful evidence about the causal effect between HT and MI. Given that MI remains the leading cardiovascular morbidity, the results promote the demand for public health concepts aimed at highlighting the importance of proper range of BP control to lower the society health burden of MI and associated complications.

Hypertension is involve in endothelial damage, hypercoagulability and cell dysfunction. The mechanisms triggering dysfunction of endothelial cells are multifactorial and include decreased vasodilator activity and increased vasoconstrictor activity (or sensitivity) (32, 33). Atherosclerosis is a gradually inflammatory disease triggered by the accumulation of sediment like Fat mass or plaques in blood vessels, resulting in arteries narrowing (33). Hypertension can trigger plaque formation, which causes a rupture in the endodermis and produces an accumulation of low-density lipoprotein (LDL) in the sub-endothelial space. Trapped LDL, which causes the expression of adhesion molecules, is subject to oxidation by reactive oxygen species (ROS), and monocytes circulating in the blood system and T lymphocytes adhere to these adhesion material and are redirected by pro-inflammatory cytokines and chemoattractants into the intima (34–36). Monocytes which differentiate into macrophages ingest oxidized LDL in an uncontrolled manner, eventually establishing the foam cells, which further perpetuate locally inflammatory responses and recruitment of cell. Then, B and T lymphocytes enter the intima, creating a vicious cycle to further stimulate macrophages. A fatty streak forms because of the eventual death of foam cells. Smooth muscle cells (SMC) remove and proliferate in the sub-endothelial area and form a cap of fibrous collagen, which causes calcification and ultimately results in the hardening of the atherosclerotic plaque (35, 36). When plaque cracks, the fibrous cap splits and the strongly thrombogenic lipid core to blood exposes (37–39). After the thrombosis, cardiomyocytes die fastly due to a variety of factors, including hypoxia and energy depletion in the hypoxia, re-oxygenation, ROS state (37). Most of myocardium without reperfusion is affected by necrosis, percutaneous coronary intervention (PCI) or pharmacologic thrombolysis within 6 h (37).

Traditional observational epidemiology is one of the most strong methods of proving the hypothesis of etiology in epidemiological study and is likely affected by confounding factors that have created many difficulties in revealing causal inference and the cause of disease. For instance, the diagnosis of HT hinges on the precise measurement of BP. As Kaplan stated, “The measurement of blood pressure is likely the clinical procedure of greatest importance that is performed in the sloppiest manner” (40). The correct diagnosis should. In accordance with several results measured on different days. Traditionally, mercury sphygmomanometers have outstanding accuracy, and electronic sphygmomanometers should be recalibrated periodically. This might cause errors in the measurements. Second, there are differences in the measurement values taken in the doctor's office and those taken at home, which is called home blood pressure (HBP) and white-coat hypertension (WCH) (32). This might explain why previous observational studies have yielded conflicting results; there are inherent limitations that are prone to several biases.

The application of MR can dexterously overcome the characteristic of traditional epidemiological study in expounding the etiology, such as confounder and unknown causal sequences, and derivative new tactics and approaches for epidemiological study in etiology (41). Another merit of conducting MR to explore causality between BP and MI is related to the troubles in engaging and carrying out clinical trials to assess the role of intensively control range of BP on the subsequent risk of MI, as such trials would incur high costs related to the large size of patients to be recruited and a long term follow-up period. The strengths of our design are related to a large research sample, which gave us a chance to conduct analysis by synthesis of MI, and the well-powered GWASs used to acquire genetic instruments for our MR analyses.

There are several limitations in our study. First, we imposed restrictions on the study population to the main individuals of European ancestry to lower bias from population stratification. This restriction imposed lowered the transferability to individuals who have other genetic backgrounds. Second, because the individual data were not available, we could not perform analyses stratified by subtypes and severity of MI. Additionally, the application of a genetic instrument including number of genetic variants for each component part of BP elevates the risk of including pleiotropic SNPs. However, we addressed horizontal pleiotropy through MR sensitivity analysis. Nevertheless, we could not address unobserved pleiotropy. When some instrumental SNPs show horizontal pleiotropy, our estimates of IVW effect are apt to be biased.

## Conclusions

Performing a genetic method, we verified that BP levels are causally associated with MI risk.

## Data availability statement

The original contributions presented in the study are included in the article/Supplementary material, further inquiries can be directed to the corresponding author/s.

## Ethics statement

Ethical review and approval was not required for this study in accordance with the local legislation and institutional requirements.

## Author contributions

Z-QY and T-TF conceived the study, participated in the design, performed the statistical analyses, and drafted the manuscript. B-LX conceived the study, participated in the design, and helped to draft the manuscript. QW revised the paper. All authors gave final approval and agree to be accountable for all aspects of work ensuring integrity and accuracy.

## Funding

This present study was supported by the Second Affiliated Hospital of Anhui Medical University – Hefei Institute of Intelligent Machinery, Chinese Academy of Sciences–Joint Research Fund for Prevention and Control of Chronic Diseases (MBLHJJ202007).

## Conflict of interest

The authors declare that the research was conducted in the absence of any commercial or financial relationships that could be construed as a potential conflict of interest.

## Publisher's note

All claims expressed in this article are solely those of the authors and do not necessarily represent those of their affiliated organizations, or those of the publisher, the editors and the reviewers. Any product that may be evaluated in this article, or claim that may be made by its manufacturer, is not guaranteed or endorsed by the publisher.
