# Supplementary material for: Causal associations between blood pressure and the risk of myocardial infarction: A bidirectional Mendelian randomization study
Source: Front Cardiovasc Med. 2022 Nov 10;9:924525. doi: 10.3389/fcvm.2022.924525 (PMC9686852; doi:10.3389/fcvm.2022.924525)
Supplement: Supplementary file 1 [file Data_Sheet_1.pdf]

Table1

	GWAS ID	Consortium	Author	sample size	number of snp	population	PMID
SBP	ieu-b-38	ICBP	Evangelou, E	757601	7088083	European	30224653
DBP	ieu-b-39	ICBP	Evangelou, E	757601	7160619	European	30224653
PP	bbj-a-46	NA	Ishigaki K	136249	6108953	East Asian	29403010
MI	ukb-d-I9_MI	UKB	Neale lab	361194	12640541	European	NA

Figure1

A

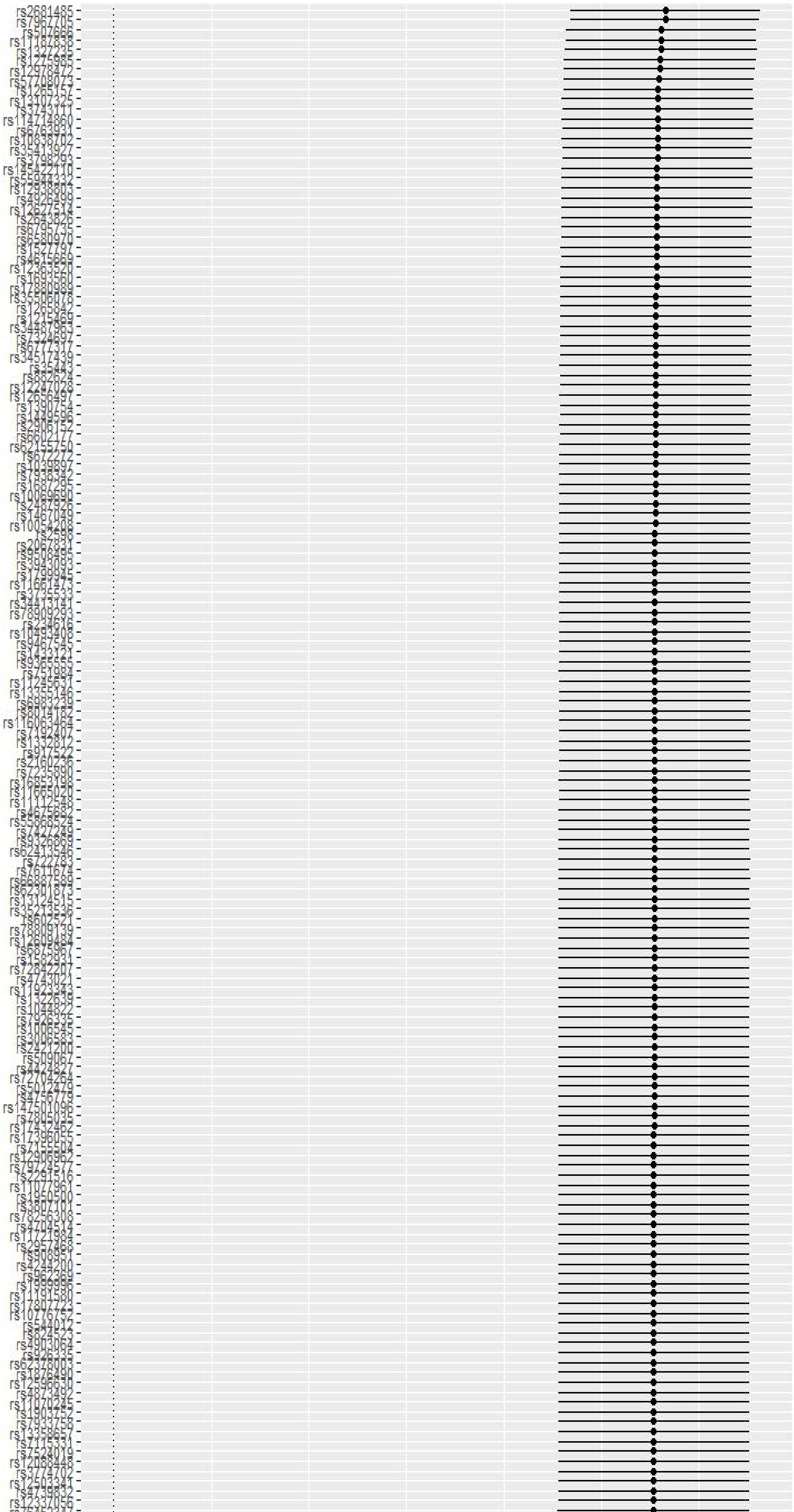

B

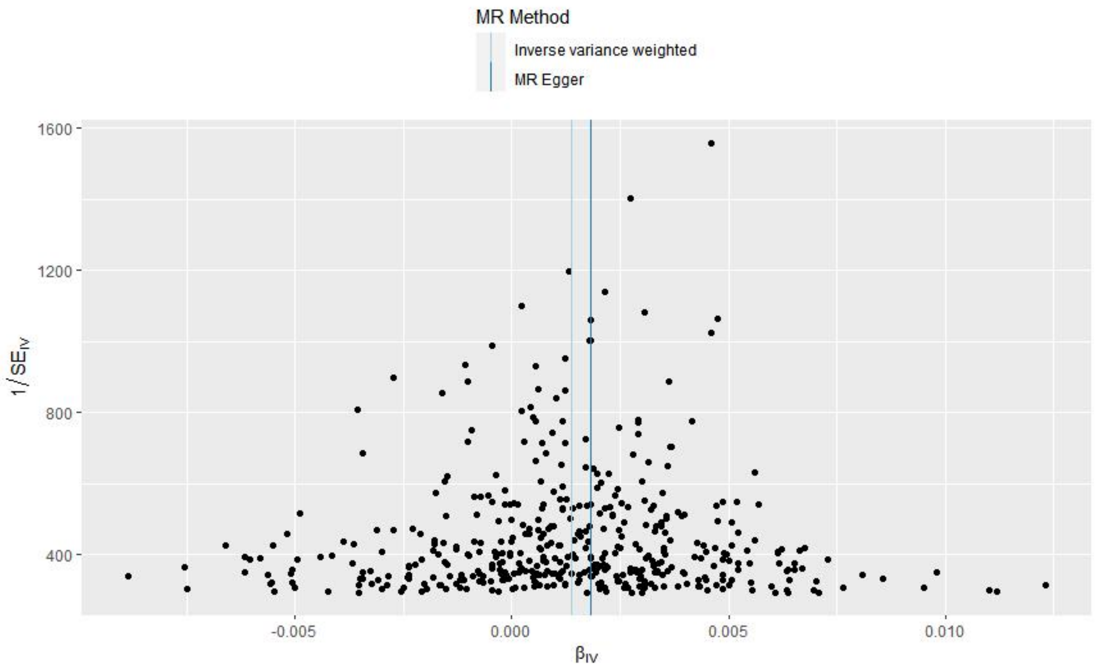

Leave-one-out sensitivity analysis and funnel plots in the DBP→MI MR analysis(A) Leave-one-out sensitivity analysis.(B) Funnel plot of the relationship between the causal effect of DBP on MI.

Figure2

A

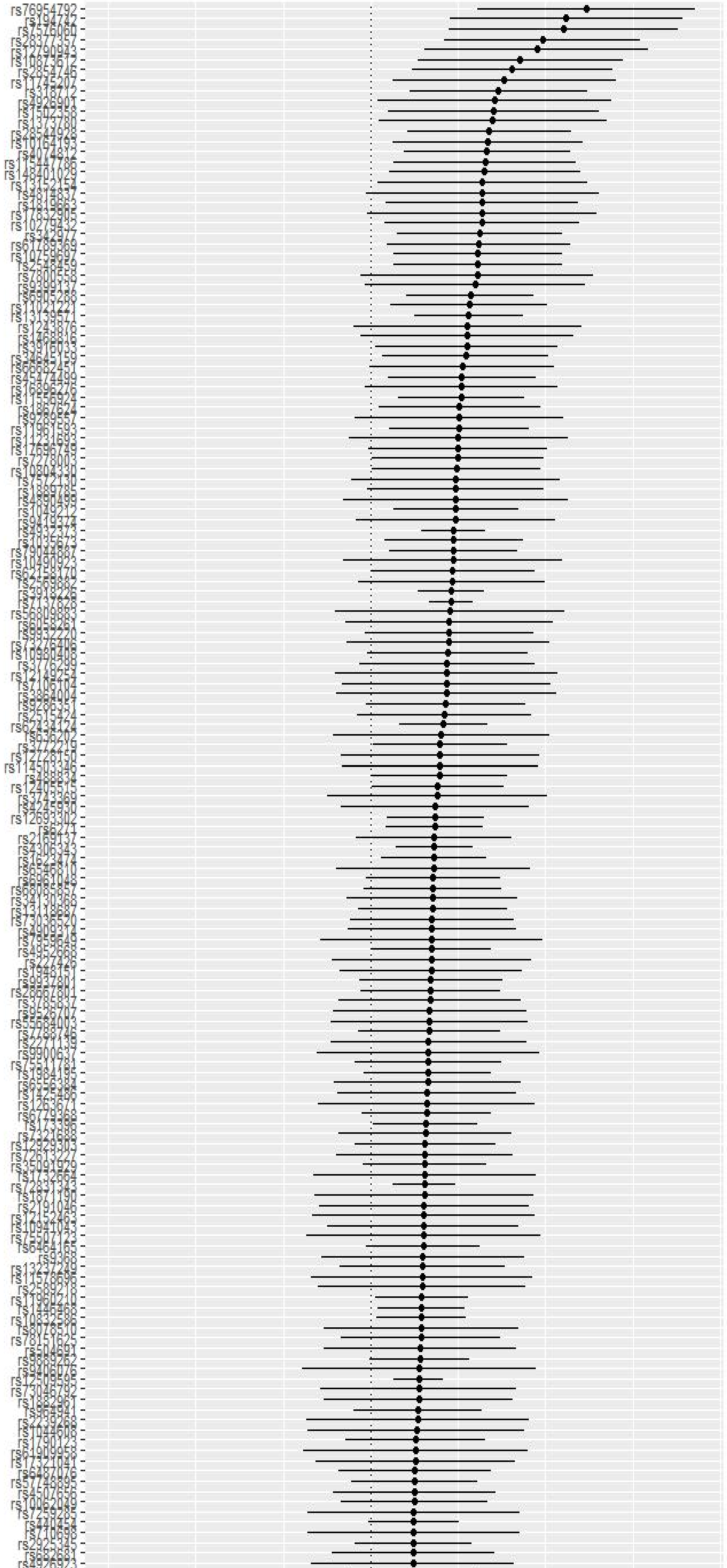
rs76954792

B

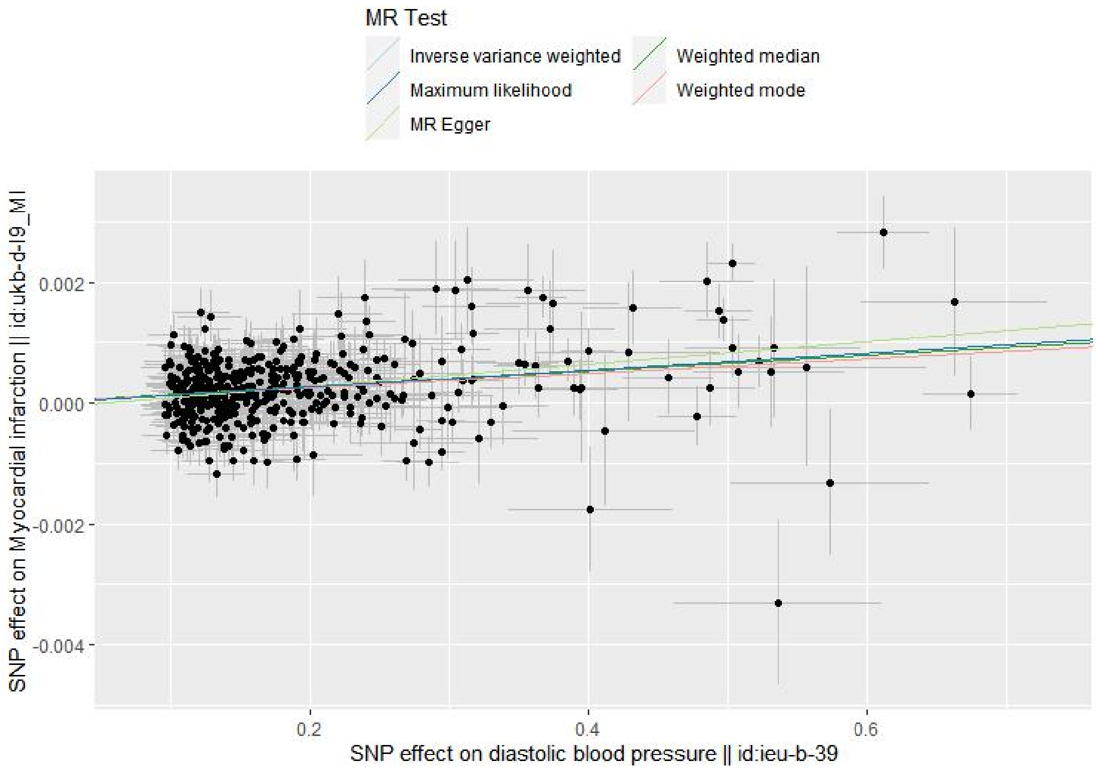

Forest plots (A) and scatter plots (B) of causal effects between DBP-associated SNPs and risk of MI. The slopes of each line in the scatter plots represent the causal association for each method.

Figure3

A

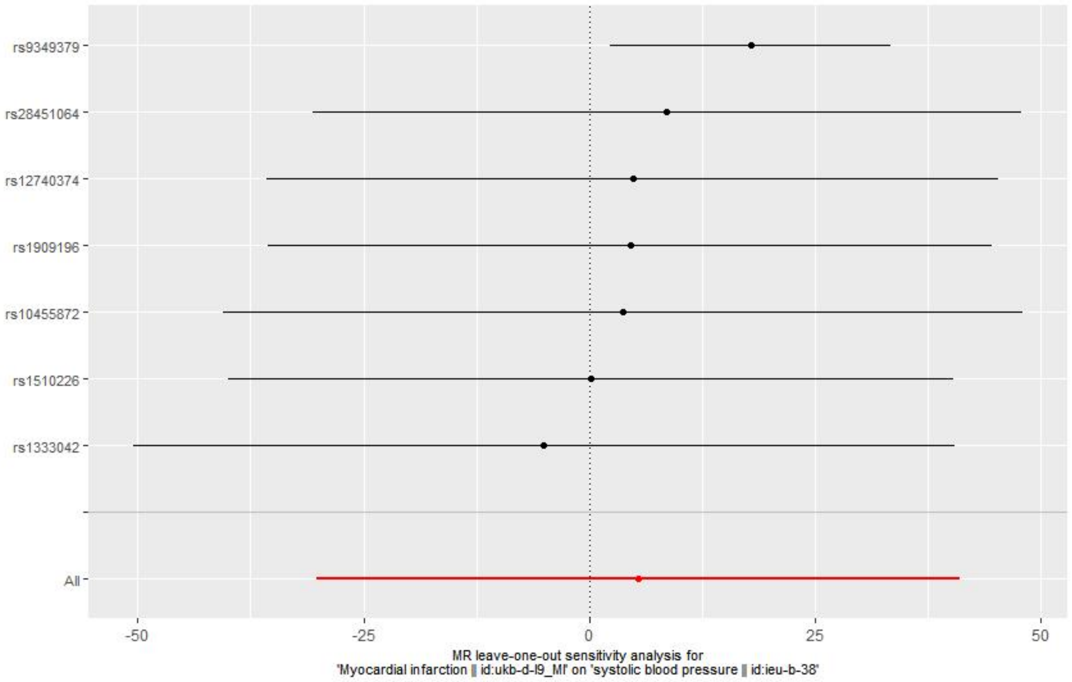

B

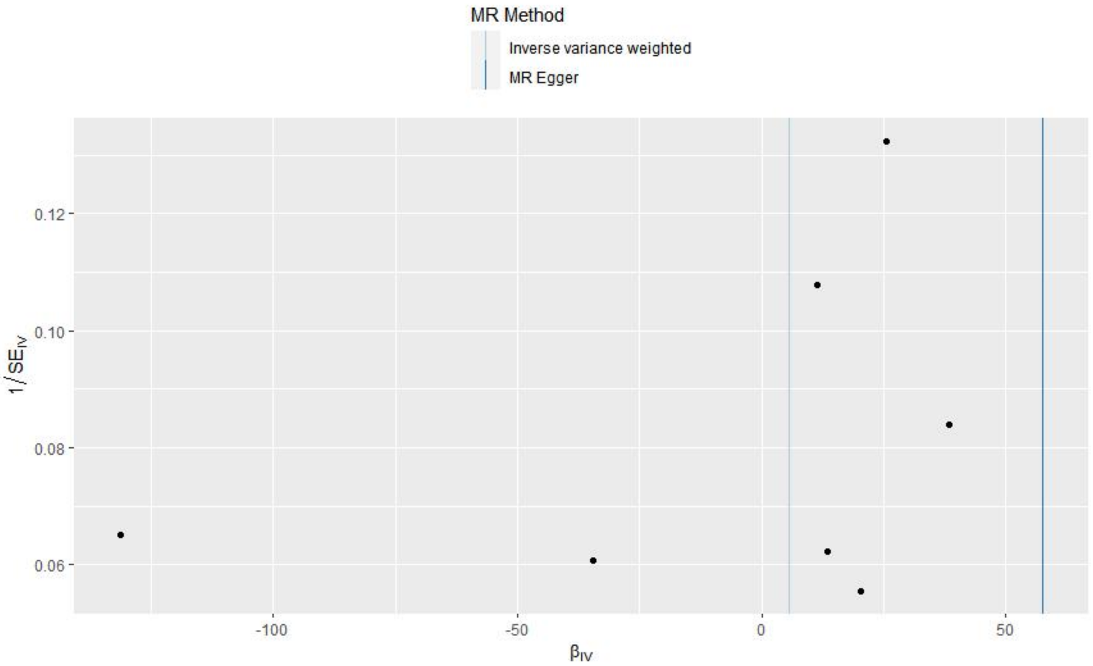

Leave-one-out sensitivity analysis and funnel plots in the MI→SBP MR analysis(A) Leave-one-out sensitivity analysis.(B) Funnel plot of the relationship between the causal effect of MI on SBP.

Figure4

A

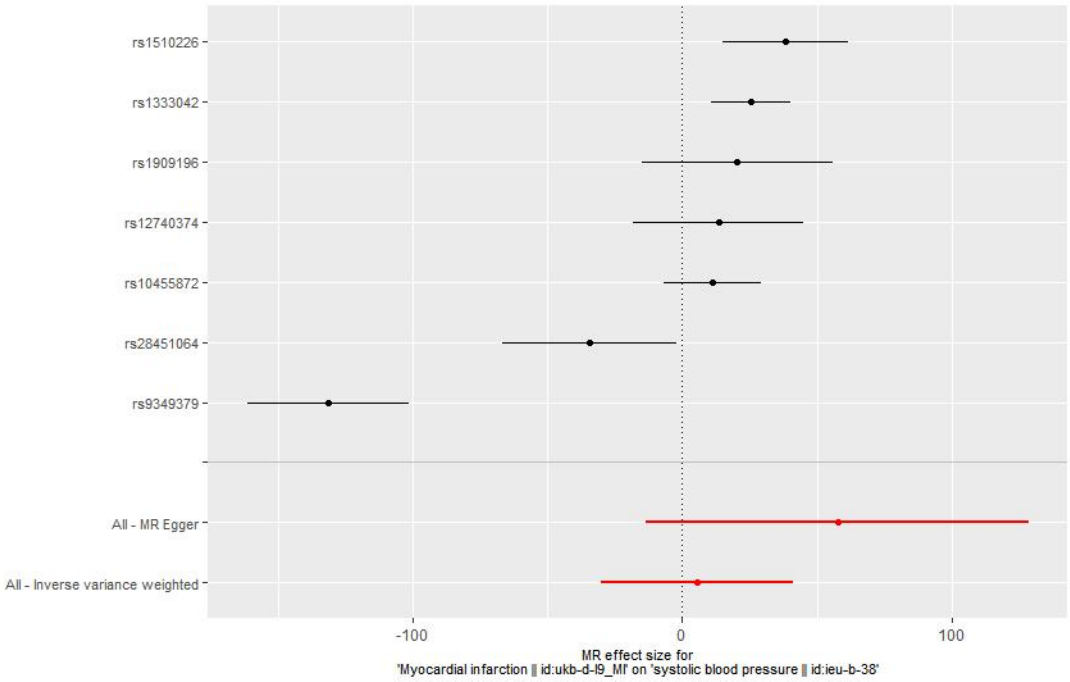

B

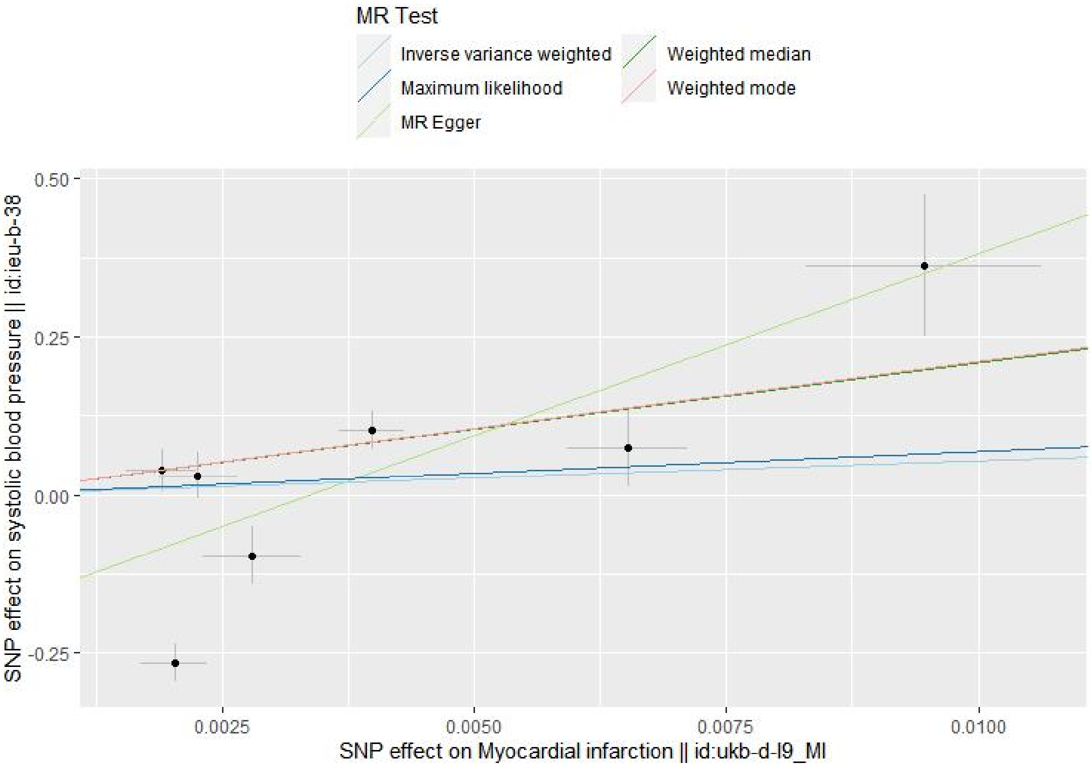

Forest plots (A) and scatter plots (B) of causal effects between MI-associated SNPs and risk of SBP.

Figure5

A

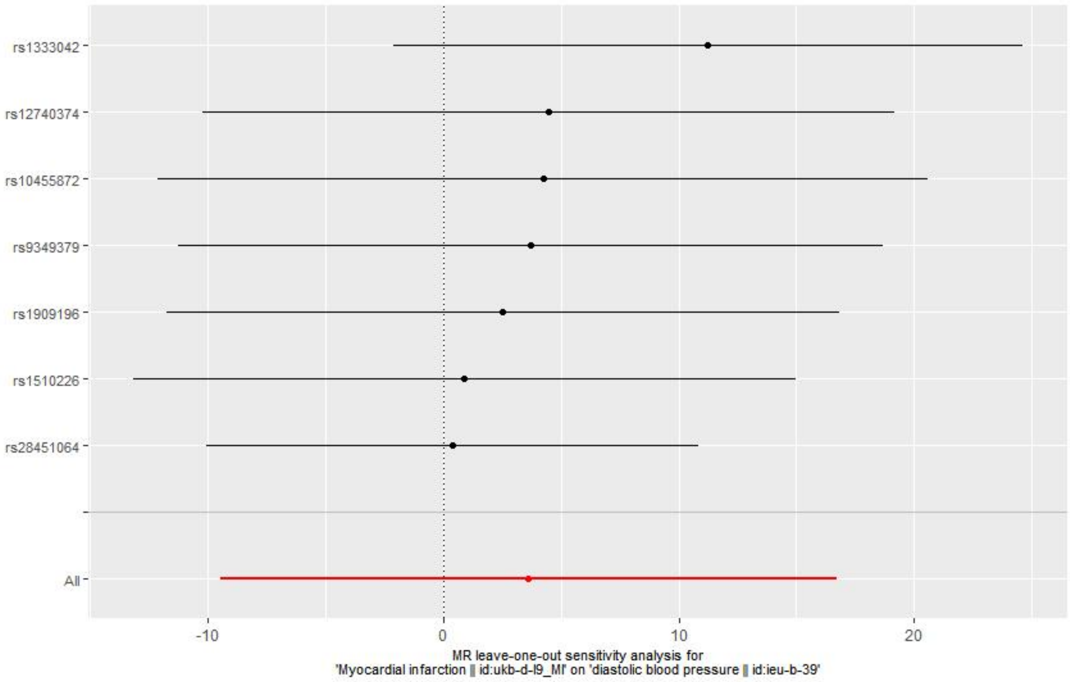

B

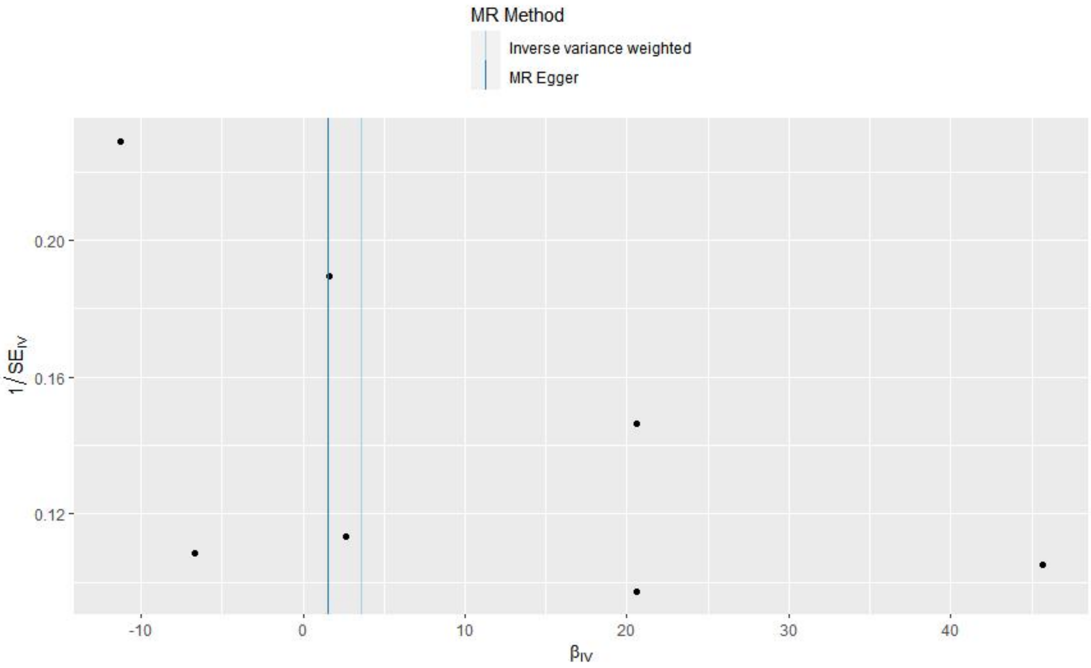

Leave-one-out sensitivity analysis and funnel plots in the MI→DBP MR analysis(A) Leave-one-out sensitivity analysis.(B) Funnel plot of the relationship between the causal effect of MI on DBP.

Figure6

A

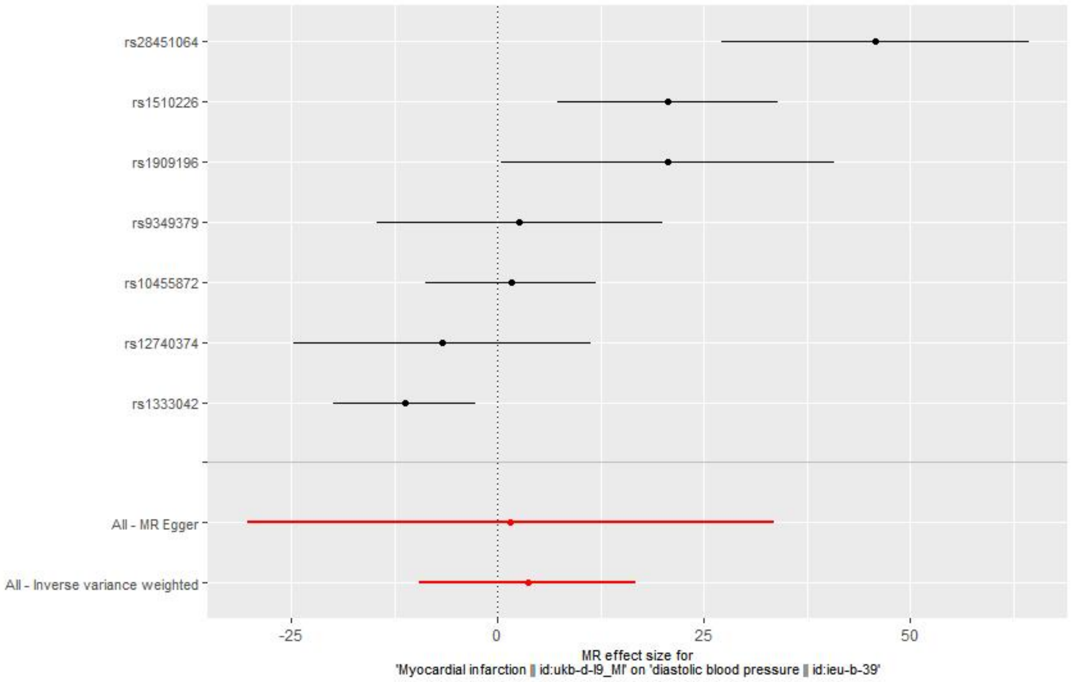

B

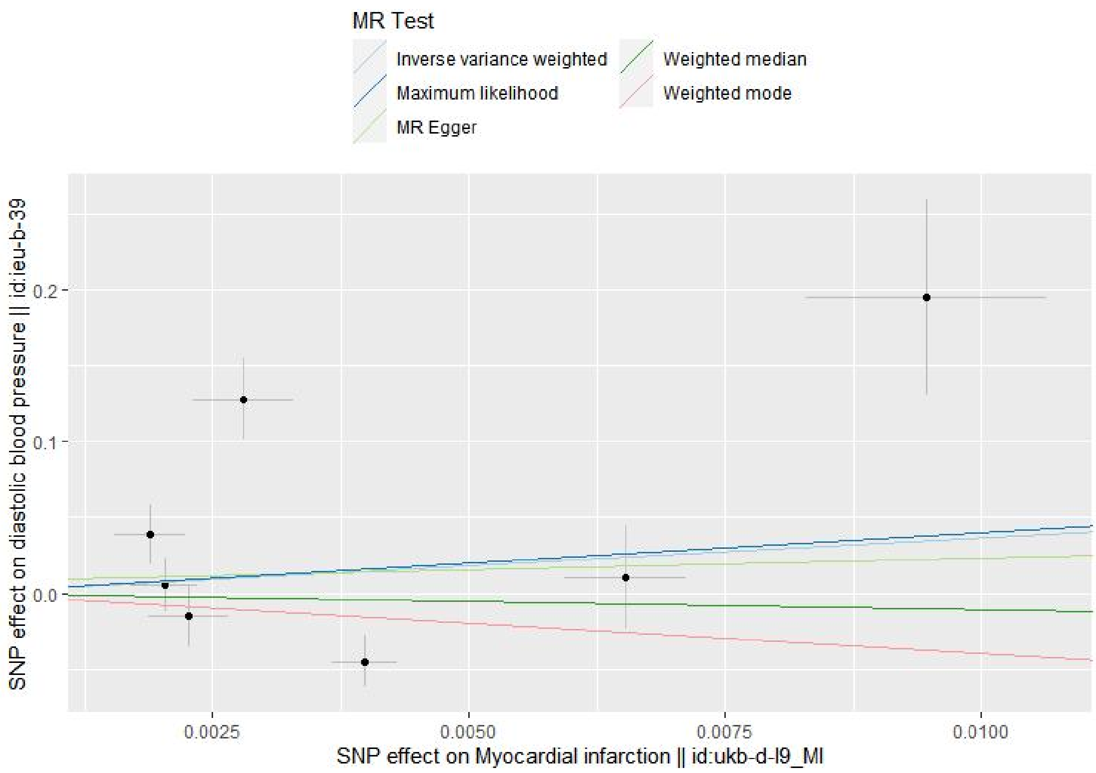

Forest plots (A) and scatter plots (B) of causal effects between MI-associated SNPs and risk of DBP.

Figure7

A

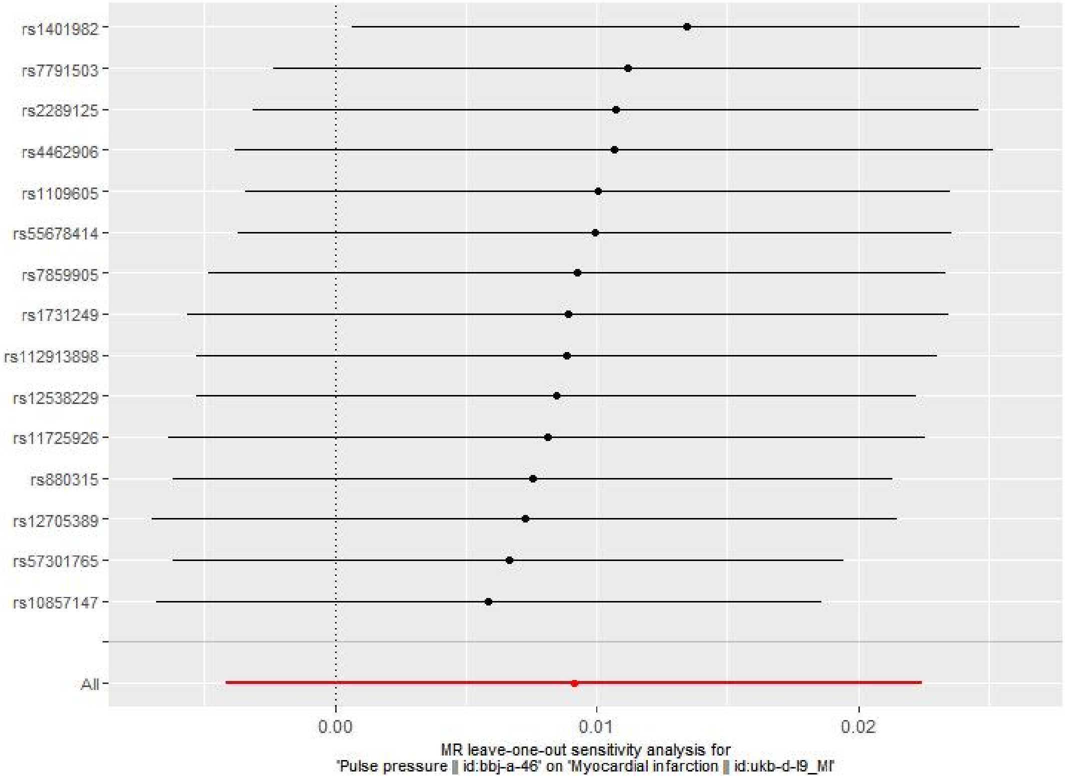
rs1401982
rs7791503
rs2289125
rs4462906
rs1109605
rs55678414
rs7859905
rs1731249
rs112913898
rs12538229
rs11725926
rs880315
rs12705389
rs57301765
rs10857147
All
0.00
0.01
0.02
MR leave-one-out sensitivity analysis for
'Pulse pressure || id:bbj-a-46' on 'Myocardial infarction || id:ukb-d-I9_MI'

B

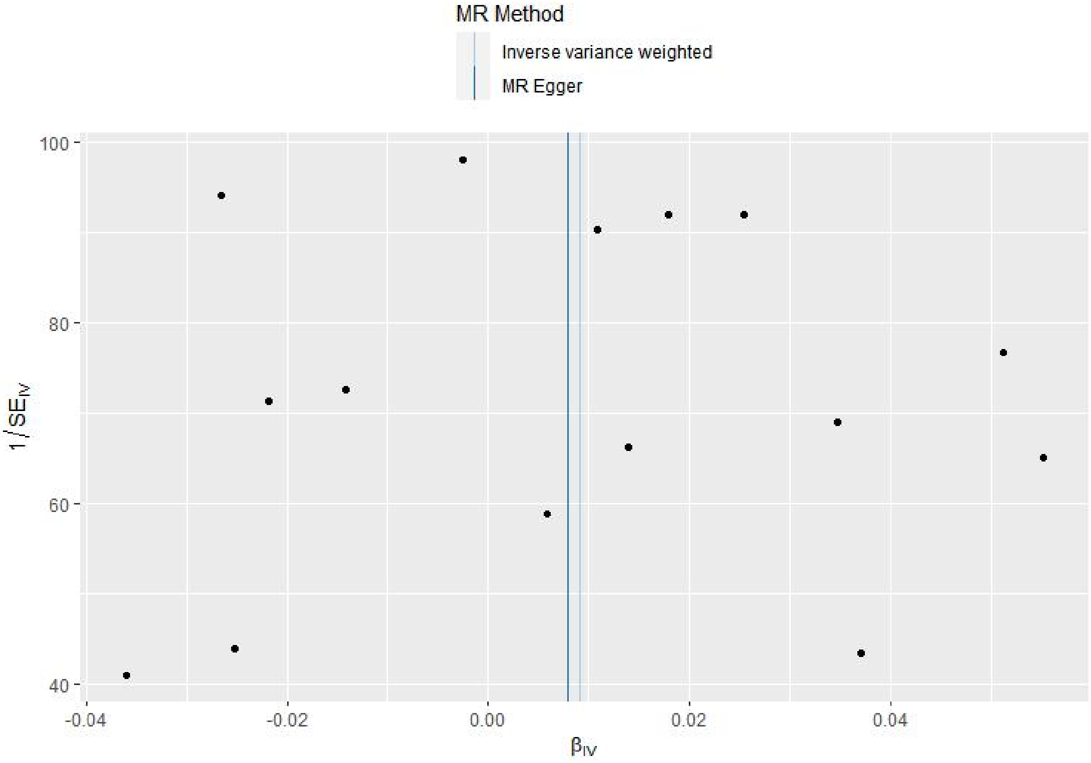
MR Method
Inverse variance weighted
MR Egger
100
80
60
40
1/SE$_{IV}$
-0.04
-0.02
0.00
0.02
0.04
β$_{IV}$

Leave-one-out sensitivity analysis and funnel plots in the PP→MI MR analysis(A) Leave-one-out sensitivity analysis.(B) Funnel plot of the relationship between the causal effect of PP on MI.

Figure8

A

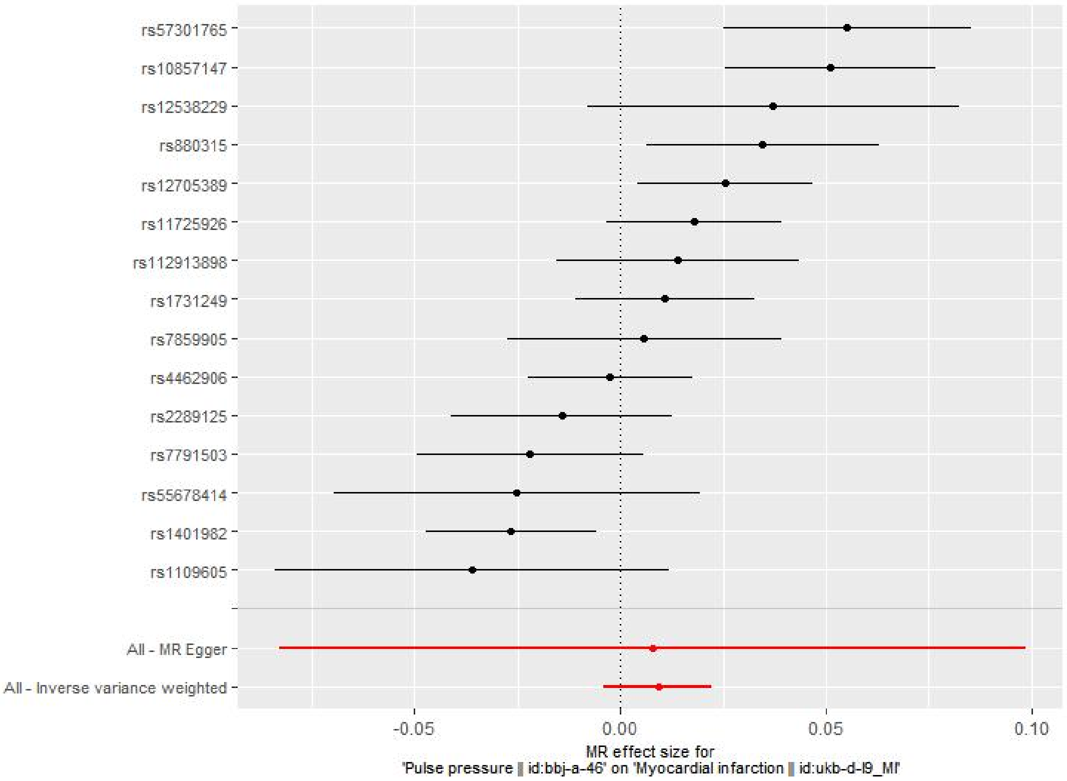

B

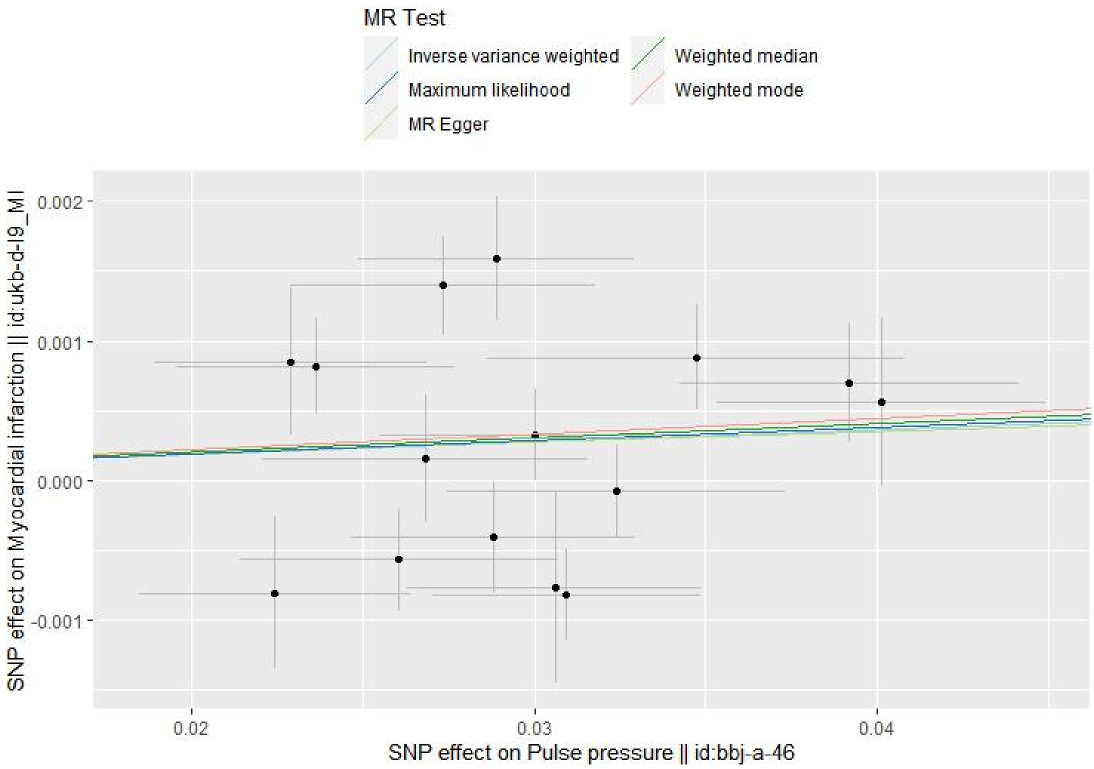

Forest plots (A) and scatter plots (B) of causal effects between PP-associated SNPs and risk of MI.

Figure9

A

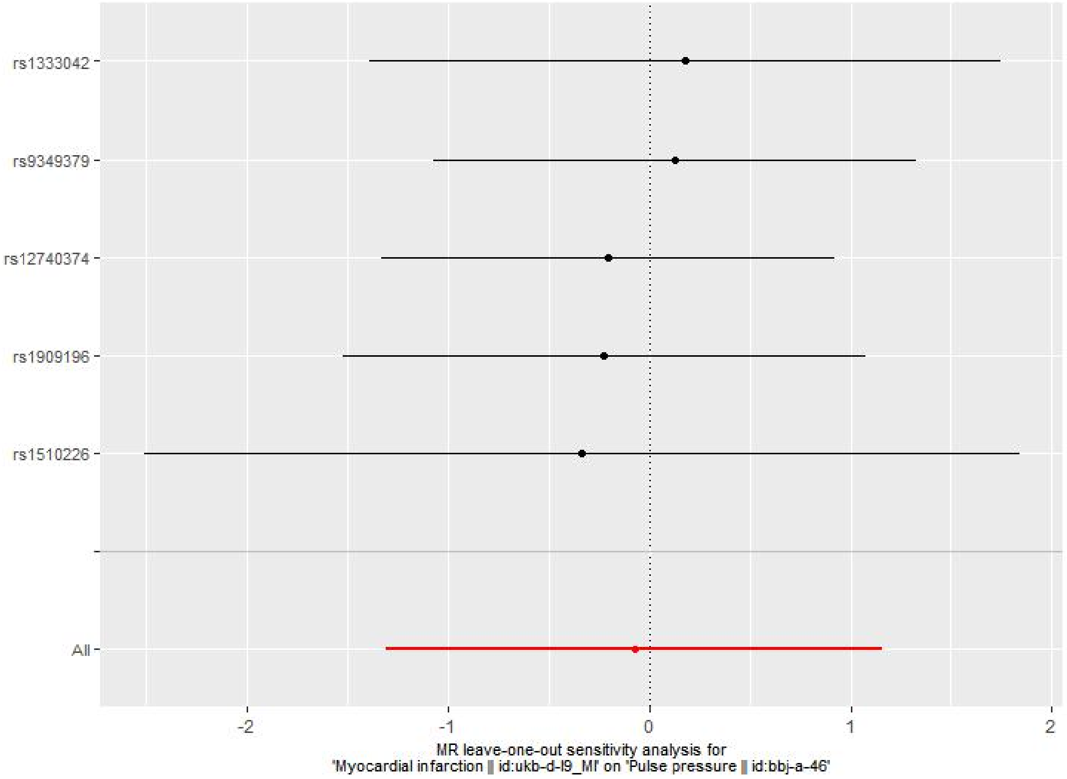
rs1333042
rs9349379
rs12740374
rs1909196
rs1510226
All
-2
-1
0
1
2
MR leave-one-out sensitivity analysis for
'Myocardial infarction || id:ukb-d-I9_MI' on 'Pulse pressure || id:bbj-a-46'

B

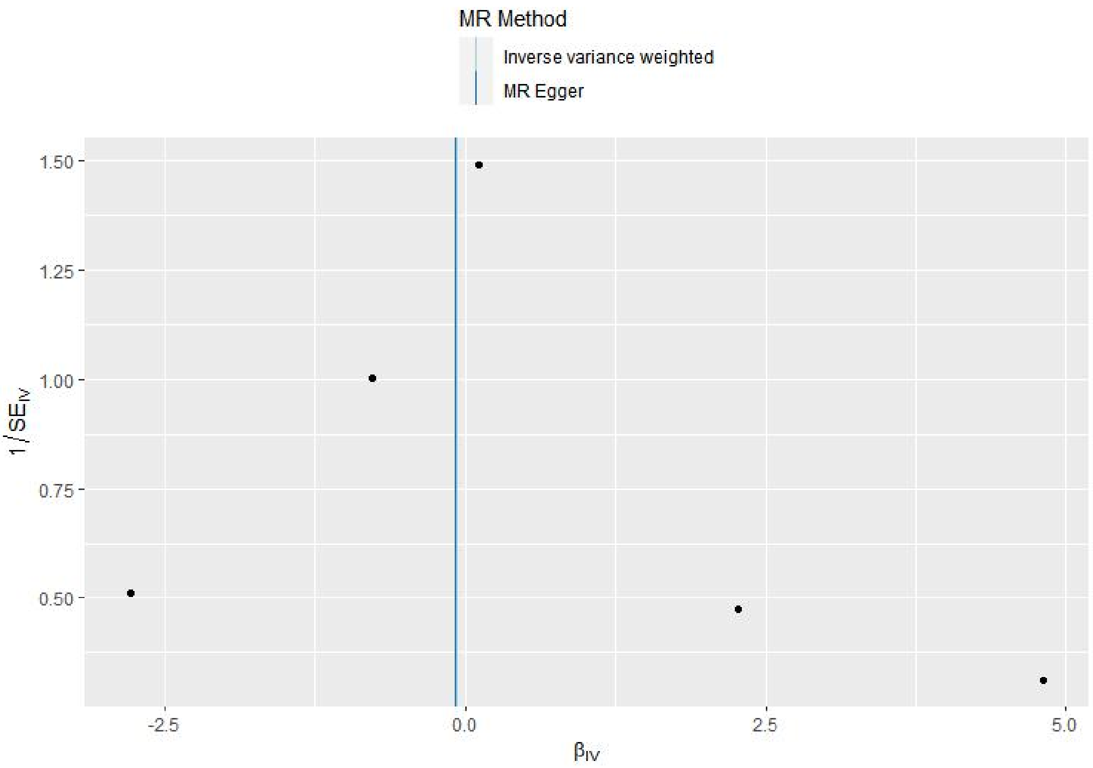
MR Method
Inverse variance weighted
MR Egger
1.50
1.25
1.00
0.75
0.50
1/SE_IV
-2.5
0.0
2.5
5.0
β_IV

Leave-one-out sensitivity analysis and funnel plots in the MI→PP MR analysis(A) Leave-one-out sensitivity analysis.(B) Funnel plot of the relationship between the causal effect of MI on PP.

Figure10

A

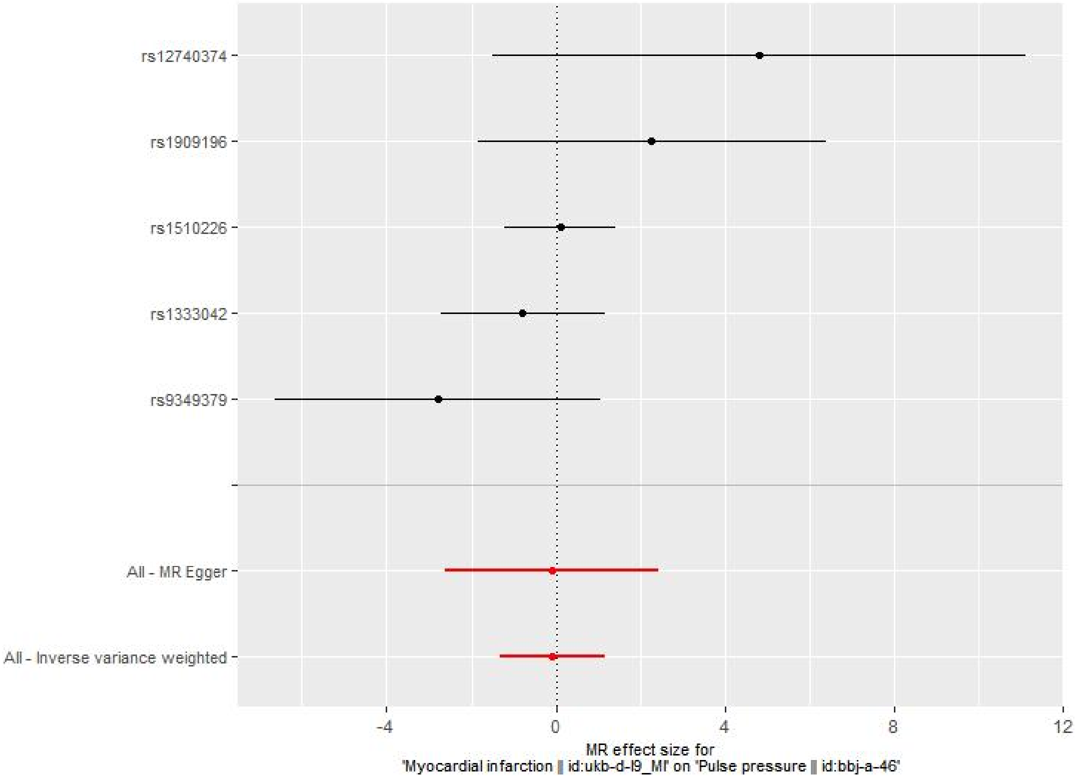

B

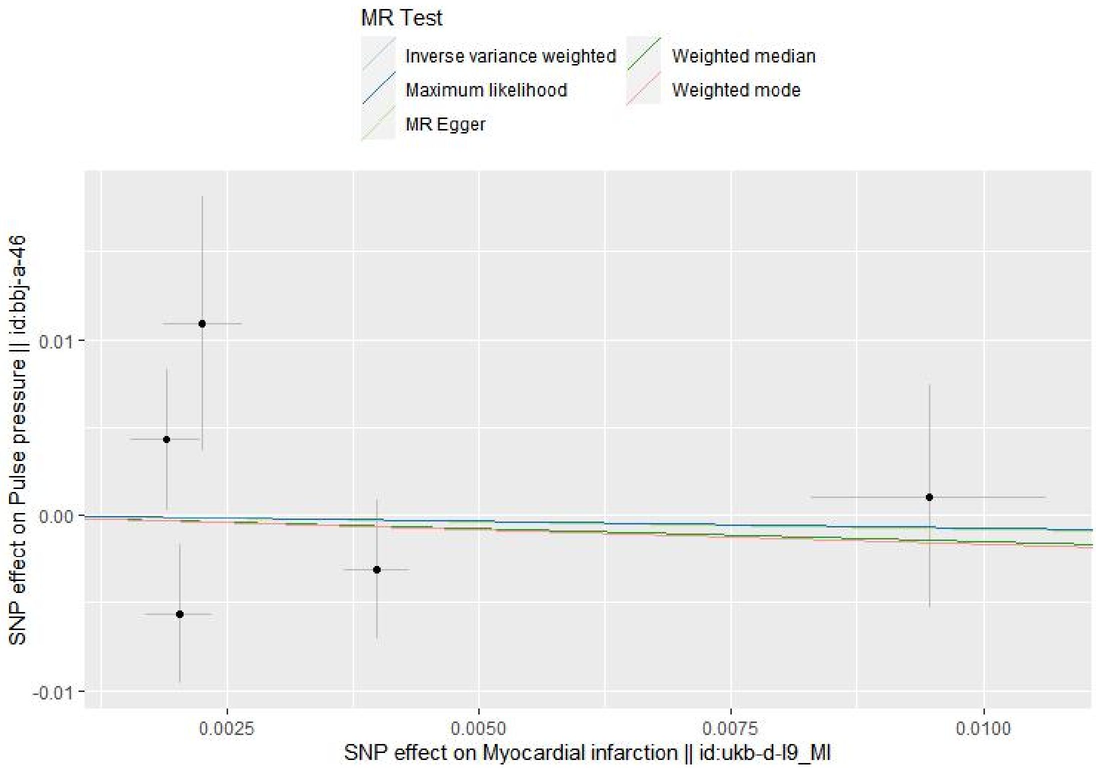

Forest plots (A) and scatter plots (B) of causal effects between MI-associated SNPs and risk of PP.
